# Behavioural Differences and Neural Substrates of Altruistic and Spiteful Punishment

**DOI:** 10.1038/s41598-017-15188-w

**Published:** 2017-11-07

**Authors:** Toshio Yamagishi, Yang Li, Alan S. R. Fermin, Ryota Kanai, Haruto Takagishi, Yoshie Matsumoto, Toko Kiyonari, Masamichi Sakagami

**Affiliations:** 1 Graduate School of International Corporate Strategy, Hitotsubashi University, Tokyo, Japan; 2 Brain Science Institute, Tamagawa University, Tokyo, Japan; 3 Department of Psychology, University of Melbourne, Melbourne, Australia; 4 Department of Neuroinformatics, Araya Inc., Tokyo, Japan; 5 School of Social Informatics, Aoyama Gakuin University, Kanagawa, Japan

## Abstract

Altruistic punishment following social norm violations promotes human cooperation. However, experimental evidence indicates that some forms of punishment are spiteful rather than altruistic. Using two types of punishment games and seven non-strategic games, we identified strong behavioural differences between altruistic and spiteful punishers. Altruistic punishers who rejected unfair offers in the ultimatum game and punished norm violators in the third-party punishment game behaved pro-socially in various non-strategic games. Spiteful punishers who rejected unfair offers in the ultimatum game but did not punish norm violators in the third-party punishment game behaved selfishly in non-strategic games. In addition, the left caudate nucleus was larger in spiteful punishers than in altruistic punishers. These findings are in contrast to the previous assumption that altruistic punishers derive pleasure from enforcement of fairness norms, and suggest that spiteful punishers derive pleasure from seeing the target experience negative consequences.

## Introduction

Altruistic punishment following social norm violations plays a critical role in promoting human cooperation^1–4^. Based on well-established findings obtained using economic games (e.g., the ultimatum game (UG) and the third-party punishment game (TPPG)), some research suggests that evolution has favoured a behavioural disposition toward altruistic punishment^2,5^. Furthermore, altruistic punishers may derive pleasure from enforcing social norms of fairness and establishing fairness in a social context^6,7^. The strong reciprocity model of the evolution of human cooperation^3,4^ has gained some acceptance partly based on the observation that unfair offers in the UG are frequently rejected with pleasure. However, recent studies have proposed an alternative view, suggesting that spiteful punishment plays an equally important role^8–12^. For example, in one study that compared participants’ choices in the UG and the dictator game (DG), altruistic punishers in the UG also behaved altruistically in the DG—a non-strategic game in which the players’ choices directly reflect their preferences rather than strategic attempts to maximise their own self-interests—whereas spiteful punishers behaved selfishly in the DG^11^. This finding suggests that spiteful punishers’ rejection of unfair offers in the UG does not stem from altruistic concerns such as fairness or social norm enforcement.

In the present study, we proposed and successfully tested the hypothesis that altruistic punishers who exert costly punishment in the TPPG would behave pro-socially in non-strategic games, regardless of their behaviour in the UG, whereas spiteful punishers who reject unfair offers in the UG but do not exert costly punishment in the TPPG would behave selfishly in non-strategic games. This hypothesis is based on the previous finding that rejections of unfair offers in the UG do not necessarily reflect altruistic concerns for social norm enforcement^8–12^. Thus, at least some rejections of unfair offers in the UG may reflect spiteful rather than altruistic concerns, the goal of which may be to reduce the relative standing of the target proposer vis-à-vis one’s own standing. One major difference between punishment in the TPPG and rejection of unfair offers in the UG is that the target of punishment in the former is a third-party with whom the punisher has not established an interpersonal relationship, whereas the target in the latter is an individual who intends to impose a status difference (i.e., difference in allocated resources) upon the punisher. This difference implies that spiteful concerns have no role in the punishment of norm violations, and thus that only altruistic concerns drive punishment in the TPPG. In contrast, those who do not exert punishment in the TPPG and yet reject unfair offers in the UG are driven by only spiteful concerns. If this predicted difference between altruistic punishers and spiteful punishers is in fact the case, altruistic punishers should behave pro-socially in other non-strategic games, whereas spiteful punishers should behave selfishly in non-strategic games.

We tested this hypothesis by examining the behaviour of 453 non-student participants from Tokyo suburbs during seven non-strategic games in which the participants behaviours were based solely on their social preferences, and two punishment games in which two types of punishment behaviour were possible. We defined altruistic punishers as those who rejected unfair offers in the UG and the TPPG in which they had not been negatively affected by the norm violator’s behaviour. Spiteful punishers were defined as those who rejected unfair offers in the UG and yet did not punish norm violation in the TPPG.

Those who punished in both games may be driven by both altruistic and spiteful concerns. Their rejection of unfair offers in the UG may reflect their concerns for altruistic norm enforcement as in the TPPG, or may reflect spiteful concerns similar to those of spiteful punishers. Our results indicated that altruistic punishers behaved pro-socially in a variety of non-strategic games, and that spiteful punishers behaved selfishly in the same non-strategic games. Those who punished in both games exhibited similar pro-social behaviour to that of altruistic punishers, and were thus categorised as altruistic punishers. In addition, their rejection behaviours in the UG appeared to reflect altruistic rather than spiteful concerns. These findings suggest that spiteful punishers who reject unfair offers in the UG but do not exert costly punishment in the TPPG anticipate pleasure from letting the unfair partner suffer, but not from achieving altruistic norm enforcement.

We then explored whether behavioural differences between altruistic and spiteful punishers are associated with distinct neural substrates. For this analysis, we conducted a magnetic resonance imaging experiment to estimate the grey matter volume (GMV) of individual cortical and subcortical brain regions. Our results indicated that the two types of punishers not only differed in their behaviour but also in GMV of the left caudate nucleus (CDn): Spiteful punishers exhibited a larger left CDn than altruistic punishers. Previous studies have implicated the dorsal striatum—which includes the CDn—in the punishment of unfair partners in two-person games similar to the UG, suggesting that the punishment of unfair partners is driven by the anticipation of pleasure^6,7^. Taken together, these our findings support the conclusions derived from the behavioural experiment of the present study: Spiteful punishers anticipate pleasure from seeing their target suffer by rejecting unfair offers, while altruistic punishers reject unfair offers as a deliberate attempt to punish norm violators (as in the TPPG), rather than seeking pleasure from enforcing fairness norms.

## Results

We analysed the behavioural and neural data of 453 participants (see Fig. S1 for the demographic data of the sample). We constructed an overall measure of pro-social behaviour using seven economic games in which behavioural choices are straightforward reflections of the players’ social preferences, uncontaminated by strategic calculations. Participants anonymously engaged in each game one time. In these games, players did not experience any repercussions on their own welfare by altering the behaviour of other players’ behaviours: simultaneously played prisoner’s dilemma games (PDG1, DG2), social dilemma games (SDG1, SDG2), dictator games (DG1, DG2), and the trust game (TG). In the TG, all participants played both the proposer’s and the responder’s role. Details regarding the games and construction of the overall measure of pro-social behaviour are provided in the Supplementary Methods and Table S1.

The punishment behaviours of participants were assessed using the TPPG and UG. The TPPG was played by three players: a dictator, a recipient, and an observer. The observer witnessed the amount of money distributed (1,500 Japanese Yen [JPY; 110 JPY ≈ US$1.00) to the recipient by the target player (the dictator) in a DG, and decided how much of their own money to spend to reduce the dictator’s earnings. The earnings of the target dictator were reduced by four times the amount the observer spent for punishment. The amount spent by observers who punished unfair dictators (i.e., those who allocated one-third (JPY 500 of 1,500) or less to the recipient) was used as an indicator of punishment in the TPPG (see Supplementary Methods for further details regarding the strategy method).

Two players played the UG: a proposer and a responder. The proposer made an offer regarding how to split the 1,500 JPY with the responder, and the responder accepted or rejected the offer, again using the strategy method (cf., Supplementary Method). If the responder accepted the proposal, both the proposer and the responder earned the proposed amount. If the responder rejected the proposal, neither earned any money. The proportion of rejections of unfair proposals (one-third of JPY 1,500 or less) by the participant when he/she played the role of a responder was used as an indicator of rejection of unfair offers in the UG.

**Figure 1:**
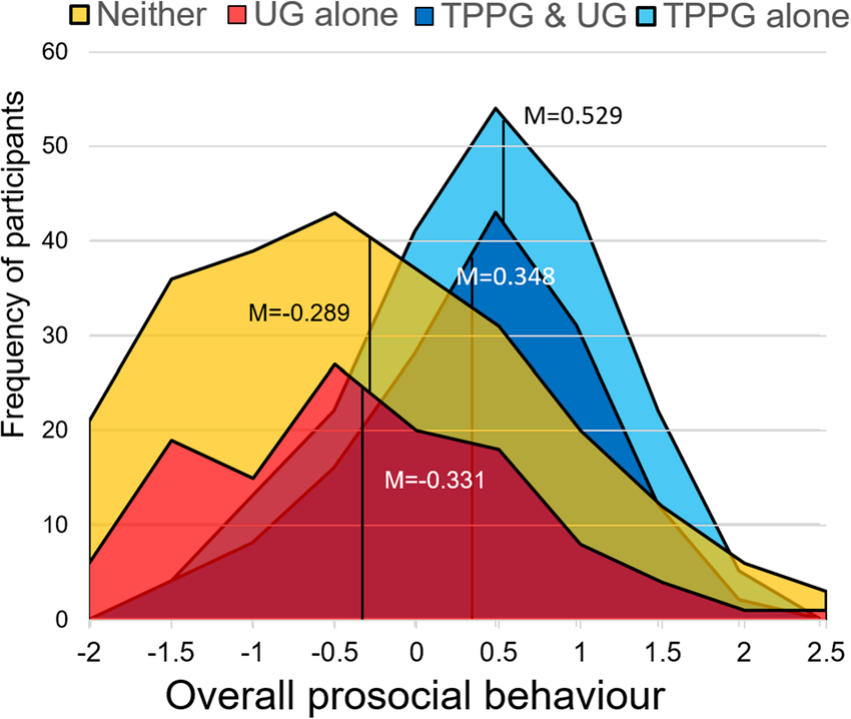
Frequency of punishers/rejecters in the TPPG and the UG as a function of overall pro-social behaviour. “Neither” refers to participants who did not exert punishment in the TPPG or reject unfair offers in the UG. “UG alone” refers to spiteful punishers who rejected unfair offers in the UG but did not punish non-cooperators in the TPPG. “TPPG alone” refers to those who exerted punishment in the TPPG but did not reject unfair offers in the UG. “TPPG & UG” refers to altruistic punishers who exerted both punishment in the TPPG and rejected unfair offers in the UG. In the figure, **“**Neither” is placed on top of “UG alone”, and “TPPG alone” is placed on top of “TPPG & UG”. The vertical axis represents the number of participants in each category for the ± 0.25 interval of the marker of overall behavioural pro-sociality (z-score).

In the TPPG, slightly less than half (45.3%) of participants spent at least some money on punishment. Among these individuals, the mean amount spent was JPY 102.276 ± 12.222 (95% confidence interval) per potential target. Participants who spent at least some money on punishment in the TPPG but did not reject unfair offers in the UG (light blue area in Fig. 1) exhibited highly pro-social behaviour (*n* = 61, *M* = 0.529 ± 0.201), and so were participants who spent some money on punishment in the TPPG and rejected unfair offers in the UG (dark blue area in Fig. 1; *n* = 144, *M* = 0.348 ± 0.121). The difference in the mean level of overall cooperation between these two groups was not significant (*t* (203) = 1.58, *P* = 0.115). We thus regarded the two types of punishers in the TPPG—those who also rejected unfair offers and those who did not reject unfair offers in the UG—as altruistic punishers. Altruistic punishers who spent at least some money on punishment in the TPPG (TPPG punishers: dark and light blue areas combined in Fig. 1) exhibited a greater level of pro-social behaviour (*n* = 205, *M* = 0.402 ± 0.104) than those who did not spend any money on punishment in the TPPG (TPPG non-punishers: red and yellow areas combined in Fig. 1; *n* = 248, *M* = −0.310 ± 0.131; *t* (442) = 8.38, *P* < 0.0001). These results support our hypothesis that altruistic punishers who exert costly punishment in the TPPG behave pro-socially in non-strategic games.

In the UG, more than half (58.1%) of participants rejected at least one unfair proposal (JPY 500 or less), and those who rejected at least one proposal rejected an average of 3.837 ± 0.146 unfair proposals (n = 5 proposals; see Supplementary Methods for details). Those who rejected at least one unfair proposal in the UG exhibited no significant differences in pro-social behaviour (UG rejecters; red and dark blue areas combined in Fig. 1; *n* = 263, *M* = 0.041 ± 0.108) from those who did not reject any unfair proposals (UG non-rejecters; yellow and light blue areas combined; *n* = 190, *M* = −0.027 ± 0.160; *t* (350) = 0.69, *P* = 0.494). However, we observed a large difference in pro-social behaviour between two types of UG rejecters—altruistic punishers who also exerted costly punishment in the TPPG (dark blue area in Fig. 1) and spiteful punishers who rejected unfair offers in the UG, but did not exert costly punishment in the TPPG (red area in Fig. 1). The pro-social behaviour of those who rejected unfair offers in the UG and exerted costly punishment in the TPPG was much higher than the overall mean of zero (*n* = 144, *M* = 0.348 ± 0.121, *t* = 5.68, *P* < 0.0001): That is, they voluntarily behaved altruistically in both games based purely on their preferences. They are altruistic punishers for whom rejection of unfair offers in the UG is driven by the same pro-social preferences that drove them to punishment in the TPPG, as well as to pro-social behaviour in other non-strategic games. In contrast, the pro-social behaviour of the spiteful punishers who rejected unfair offers but did not exert costly punishment in the TPPG was much lower than the overall mean of zero (*n* = 119, *M* = −0.331 ± 0.168, *t* = 3.90, *P* < 0.001). Moreover, the difference in pro-social behaviour between the two types of punishers was also significant (*t*(223) = 6.48, *P* < 0.0001). These results support the hypothesis that spiteful punishers behave selfishly in non-strategic games.

In the post-experimental questionnaire for the one-shot PDG (PDG2), participants evaluated how satisfied they would be if the outcome of the game was each of the four possible combinations of their own choice and the partner’s choice. The spiteful punishers exhibited a preference for the outcome of unilaterally exploiting a cooperative partner in a one-shot PDG: Their mean response to the preference for this outcome was significantly higher than that of altruistic punishers, even after controlling for their preferences for the other three outcomes (*β* = 0.607, *t*(246) = 2.76, *P* = 0.006; see Supplementary Analysis for details). That is, spiteful punishers prefer to exploit a cooperative partner in a one-shot PDG, while altruistic punishers exhibit less of a preference for exploiting a cooperative partner. These findings are in accordance with previous results, which have consistently indicated that altruistic preferences do not explain the punitive behaviours of spiteful punishers. Rather, their rejection behaviours are driven by non-altruistic, competitive goals, suggesting that unfair offers in the UG constitute a threat to their status relative to the other participa^8–12^.

**Figure 2:**
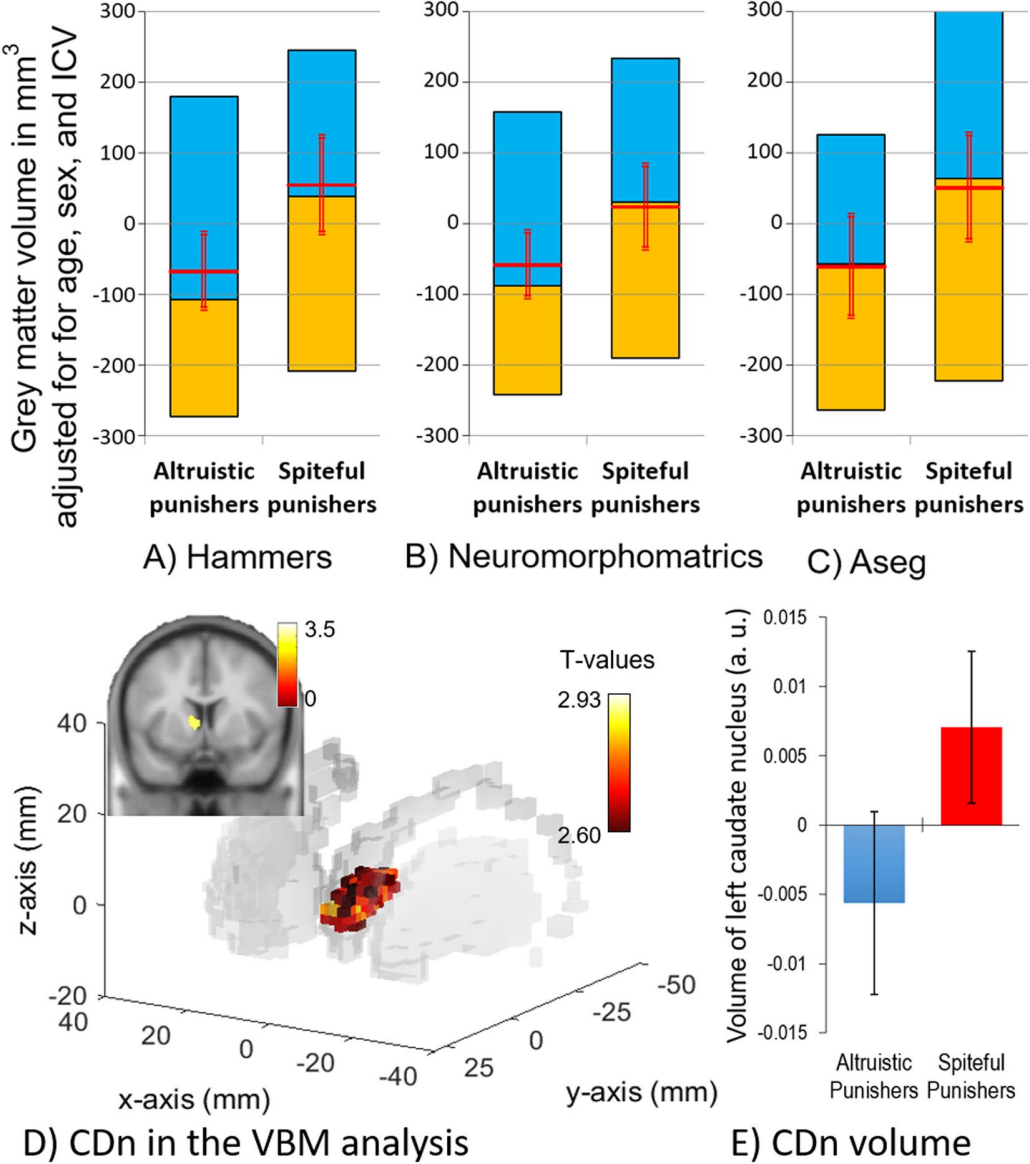
Difference in grey matter volume (GMV) of the left caudate nucleus (CDn) between altruistic and spiteful punishers based on the Hammers Atlas (Panel A), the Neuromorphometrics Altas (Panel B), the Aseg Atlas (Panel C). The caudate nucleus was identified via a whole-brain VBM analysis. The voxels identified within the CDn are presented in Panel D, while the volumes of the left CDn in spiteful punishers and altruistic punishers are presented in Panel E. In Panels A, B, and C, the GMV (in mm^3^) was adjusted for the participant’s age, sex, and intracranial volume. The yellow bar represents the second quartile, the blue bar the third quartile, the red horizontal line the mean, and the red vertical line the 95% confidence interval of the mean GMV. In Panel D, the peak voxel (peak voxel: x = −6, y = 8, z = 12; puncorrected 0.005, small-volume corrected pSVC-FWE-corrected 0.05 with 10 mm sphere radius) was significantly larger in spiteful punishers than in altruistic punishers. The voxels identified within the CDn were extracted and overlaid on a 3D projection of the structure of the basal ganglia (CDn and putamen; Panel D). The hot bar indicates the signal intensity T-values within the cluster. In Panel E, error bars indicate the 95% confidence interval.

We then examined whether behavioural differences between the altruistic and spiteful punishers are associated with a neuroanatomical substrate. We conducted a magnetic resonance imaging experiment to acquire neuroanatomical data using two parcellation methods (FreeSurfer, http://surfer.nmr.mgh.harvard.edu/ and CAT12, http://dbm.neuro.uni-jena.de/cat/) to estimate the GMV of individual brain regions specified in four different brain atlases (i.e. Hammers^13^, Neuromorphometrics^14^, Destrieux^15^, and Aseg^16^). Across the three atlases in which subcortical regions could be estimated (Hammers, Neuromorphometrics, and Aseg), we observed that only the GMV of the left CDn—which has been implicated in punishment behaviour in previous studies^6^—was consistently and significantly different between spiteful punishers (i.e., those who rejected unfair offers in the UG but did not punish in the TPPG) and altruistic punishers (i.e., those who rejected unfair offers and punished in the TPPG). The GMV of the left CDn was larger in spiteful punishers than in altruistic punishers across all atlases (Fig. 2; *β* = −96.75, *t* (225) = 2.10, *P* = 0.037 in Hammers; *β* = −85.15, *t* (225) = 2.12, *P* = 0.035 in Neuromorphometrics; *β* = −109.91, *t*(225) = 2.02, *P* = 0.044 in Aseg). Participant age, sex, and intracranial volume were controlled in this and the following analyses. The difference in CDn GMV between altruistic and spiteful punishers was also confirmed via volumetric-based morphometry (VBM) (Fig. 2D and E; see Supplementary Analysis for details).

In contrast, when the GMV of the UG punishers (including both the punishers/rejecters and non-punishers/rejecters; the dark blue and red areas in Fig. 1, respectively) was compared with that of the UG non-punishers (the non-punishers/non-rejecters and punishers/non-rejecters; light blue and orange areas in Fig. 1, respectively), no significant difference was observed (*β* = −65.76, *t* (400) = 1.87, *P* = 0.062 in Hammers; *β* = −54.54, *t* (400) = 1.79, *P* = 0.075 in Neuromorphometrics; *β* = −27.79, *t* (400) = 0.67, *P* = 0.501 in Aseg).

Finally, the left nucleus accumbens (NAc) was also larger in spiteful rejecters (red area in Fig. 1) than in altruistic rejecters (dark blue area in Fig. 1) according to the Hammers (*β* = −9.38, *t*(225) = 2.04, *P* = 0.043) and Neuromorphometrices atlases (*β* = −10.99, *t*(225) = 2.09, *P* = 0.038), but not according to the Aseg atlas (*β* = −23.36, *t*(225) = 1.57, *P* = 0.119). However, for the Aseg atlas, the right rather than left NAc was larger in spiteful punishers (*β* = −30.06, *t*(225) = 2.80, *P* = 0.006). No consistent differences in the GMV of cortical regions were observed across the three atlases or methods when values were compared between altruistic and spiteful punishers.

Notably, neither CDn nor NAc volume correlated with the level of pro-social behaviour *per se* (left CDn—Hammers *r* = −0.080, *P* = 0.106; left CDn—Neuromorphometrics *r* = −0.081, *P* = 0.106; left CDn—Aseg *r* = −0.027, *P* = 0.583; left NAc—Hammers *r* = −0.040, *P* = 0.424; left NAc—Neuromorphometrics *r* = −0.041, *P* = 0.410; right NAc—Aseg *r* = −0.036, *P* = 0.469). This finding indicates that the differences in GMV between the two types of punishers are not a simple reflection of the difference in general pro-social preferences. Rather, the differences are specific to the pro-social and spiteful nature of the rejection of unfair offers.

## Discussion

The CDn and NAc, together with their dopaminergic inputs from the midbrain, play a pivotal role in reward processing^6,7,17,18^. In previous studies^6,7^, activation of the dorsal striatum was observed in players who spent more money to deliver punishment to unfair players, suggesting that such attempts to punish are driven by the anticipation of pleasure following the enforcement of social norms and re-establishment of fairness. However, these previous studies failed to distinguish between altruistic punishment and spiteful punishment, and all punishments were instead regarded as altruistic norm enforcement. Our finding that the volumes of the CDn and NAc are larger in spiteful punishers than in altruistic punishers is the first to suggest that the pleasure anticipated in rejecting unfair offers is unique to spiteful punishment. In this case, the anticipation of pleasure is derived from letting the target suffer, in contrast to the anticipation of pleasure associated with altruistic punishment, which occurs due to enforcing social norms and establishing fairness.

For spiteful punishers, the UG may represent the adaptive domain of dominance hierarchy rather than the social exchange that most economic games are assumed to represent^8–12^. Commitment to credible reputational deterrence against external attempts to impose a lower status plays a critical role in the domain of dominance hierarchy^8–12^. We speculate that spiteful punishers tend to perceive social interactions as instances of social competition rather than social exchange and derive hedonic satisfaction from asserting their superiority against perceived competitors. Several studies have demonstrated that behavioural learning and the repetition of specific behaviour patterns leads to increased activity in brain regions whose functions are important for such behaviours, and that the recurrent use of such functions also leads to structural plastic changes in those brain areas^19,20^. Therefore, processes associated with neural plasticity may lead to enlargement of the CDn in spiteful punishers. However, direct evidence that increases in the size of the CDn and NAc indicate stronger activation and functional roles of these nuclei in the execution of punishment has yet to be established. Although some studies have reported a positive association between GMV or thickness in brain regions such as the dorsolateral prefrontal cortex^21^, temporo-parietal junction^22^, and dorsomedial/dorsolateral striatum^19^, further studies are required to verify these effects.

Many social interactions, including economic games, involve both aspects of social exchange—in which fairness plays a critical role—and aspects of status competition, in which establishing dominance plays a critical role^8–10,12,23^. However, most neuroscientific studies have compared UG non-rejecters with rejecters irrespective of the two types of punishment^6,7,24^. This rather standard practice can lead researchers to potentially biased conclusions. For example, we could have concluded that UG rejecters are no more or no less prosocial than non-rejecters based on either behavioural or neuroanatomical findings if we had failed to distinguish the two types of punishers. Furthermore, conclusions regarding neural substrates of rejecters in the UG may depend on the punisher type that dominates the specific sample used in a particular study. This oversight may be responsible for the inconsistencies concerning the impulsive or deliberate nature of cooperation and punishment^25^. Some studies have suggested that rejection in the UG represents altruistic norm enforcement, which requires the control of selfish impulses for money^24,26,27^. However, other studies supports the view that rejection in the UG represents a form of aggression that takes place when it escapes self-control^8–12^. These seemingly inconsistent findings may be avoided if future studies regarding the UG provide information that can discern altruistic from spiteful punishers, such as the participant social value orientation^28^ or behaviour in other non-strategic games that can be used to reflect a player’s pro-social preferences^11^.

The findings of the present study demonstrate that the tendency to punish for spiteful concerns, which was as prevalent as the tendency to punish for altruistic concerns in our sample, does not reflect an individual’s general pro-social preference. This finding challenges the fundamental assumption of the strong reciprocity model of the evolution of cooperation^3,4^, which assumes that the same altruistic preference is responsible for both cooperation and punishment. While spiteful punishment may lead human communities to destabilisation^29^, it may also promote fair distribution of resources by those who anticipate spiteful punishment from others^30^. The role that spiteful punishment plays in the evolution of cooperation is a relatively overlooked topic that deserves due attention in future studies.

## Methods

### Sample

Six hundred non-student residents living in and around Machida, a suburban city of Tokyo, were selected from a list of approximately 1,670 applicants who responded to a brochure that had been distributed to roughly 180,000 households. The 600 candidate participants were randomly selected from these applicants within each of five gender-specific blocks of 10-year age ranges (20–29 block to 50–59 block, each consisting of 75 men and 75 women). However, when two or more selected participants had the same address, only one of these participants was randomly selected to avoid the influence of shared experience and advice regarding strategies to be used in the games. Of the 600 who were invited to participate in the study, 564 (F = 290, M = 273) actually participated in the initial wave of the study (May – July 2012), in which demographic data were collected. One participant responded inconsistently to the demographic items, and was thus excluded from the later stages of the study. Of the remaining 563 participants, we analysed the data of 453 participants who participated in at least four of the seven economic games that we used to measure the overall level of pro-social behaviour. All included participants also participated in both the UG and the TPPG. See Fig. S1 for the demographic constitution of the sample.

### Procedure

Four to ten people participated in each session, although participants remained unaware of the number of participants in the laboratory, which consisted of 10 private compartments. Participants were placed in individual compartments in which they were completely visually isolated from the others. Instructions were displayed on each participant’s computer screen, and the participants read the instructions at their own pace. These instructions were also written using animated cartoons and, except for those regarding the PDG1, vocalised to facilitate an intuitive understanding of the game.

### Games included in the measure of overall pro-social behavior

The overall level of pro-social behaviour was assessed based on participant behaviours in seven economic games that were conducted in a one-shot situation involving no room for strategic considerations and no possibility of sanctioning: two prisoner’s dilemma games (PDG1, PDG2), two n-person social dilemma games (SDG1, SDG2), two dictator games (DG1, DG2), and one trust game (TG; only the responder’s choice). Behaviours in each game were strongly correlated with one another (Table S1). A factor analysis (principal factor method) of behaviours in these seven games (n = 358 participants who played all seven games) indicated a clear one-factor structure: The eigenvalue of the first factor was 3.629, while that of the second factor was 0.407 (Table S1). We used data from the 453 participants who played at least four of the seven games to construct the overall measure of pro-social behaviour. Each participant’s pro-social behaviour was first standardised within each game with a mean of zero and an SD of 1. Then, the mean of the standardised game scores was used as the overall score for pro-social behaviour. The overall behavioural pro-sociality score was standardised again with a mean of zero and an SD of 1 to facilitate the interpretation of the score. Details of each game are presented in the Supplementary Methods.

### Punishment games

To measure participants’ punitive behaviour, we employed the two most commonly utilised games of punishment: the UG and the TPPG. The details regarding these two games are presented in the Supplementary Methods.

### Magnetic resonance imaging data acquisition

Magnetic resonance (MR) images were obtained using a 3 Tesla Siemens Trio A Tim MRI scanner. High-resolution anatomical images were acquired using a T1-weighted 3D magnetization prepared rapid acquisition gradient echo (MPRAGE) sequence (TR = 2000 ms; TE = 1.98 ms; field of view = 256 × 256 mm; number of slices = 192; voxel size = 1 × 1 × 1 mm; average = 3 times).

### MRI analysis

T1-weighted images were used to perform brain parcellation and estimate the volume of individual brain areas based on two methods and using the available brain atlases for each method. A whole-brain voxel-based morphometry (VBM) analysis was also performed to further explore the relationship between punishment behaviour and brain structure. The details of the methods, brain atlases, and VBM analysis are described below.

### Method 1: FreeSurfer

Brain parcellation and estimation of GMV in cortical and subcortical regions were performed using a fully automated segmentation procedure implemented in FreeSurfer version 5.1.0. (http://surfer.nmr.mgh.harvard.edu/). In FreeSurfer’s automated procedure, non-brain tissues were removed using a hybrid watershed/surface and deformation procedure^31^, following which the image was affine transformed to the Talairach space. Subcortical structures were segmented and labelled based on voxel intensity and spatial position. The details of the subcortical segmentation methods are described in Fischl *et al*.^16^. In the FreeSurfer pipeline, the volumes of subcortical brain regions such as the CDn and NAc were estimated based on the Aseg brain atlas^16^, while the volumes of cortical brain regions were estimated based on the Destrieux brain atlas^15^.

### Method 2: Computational Anatomy Toolbox (CAT12)

The T1-weighted images were processed and analysed using the Computational Anatomy Toolbox (CAT12, http://dbm.neuro.uni-jena.de/cat/) and Statistical Parametric Mapping software (SPM12, http://www.fil.ion.ucl.ac.uk/spm). The images were bias-corrected, tissue-classified (GM, white matter (WM) and cerebral spinal fluid (CSF)), and registered using linear (12 parameter affine) and non-linear transformations (warping) within the CAT12 default pre-processing pipeline. This initial step generated modulated normalised data that were then smoothed via the standard SPM12 smoothing pipeline with a full-width at half maximum smoothing kernel of 8 × 8 × 8 mm. The overall GMV, WMV, CSF volume, and total intracranial volume (TIV) were then obtained using the CAT12 estimating TIV function.

CAT12 automatically performs brain structure parcellation and estimates tissue volumes of regions of interest (ROI) during the pre-processing step, separated for each participant. CAT12 uses three brain atlases for volume estimation in cortical and subcortical brain areas: the Neuromorphometrics brain atlas, LONI probabilistic brain atlas (LPBA40), and Hammers brain atlas. The details and references to these atlases can be found within the CAT12 toolbox description. The estimated ROI volume values of each participant were then combined using the CAT12 “estimate mean values inside the ROI” function. These values were used to perform the statistical analyses of the present study.

### Method 3: Voxel-based morphometry (VBM) analysis

*T*-tests were used to examine the difference in brain volume between altruistic and spiteful punishers. In this model, sex and TIV were used as covariates. Whole-brain analysis revealed that the left CDn (x = −6, y = 8, z = 2,) was significantly larger in spiteful punishers than in altruistic punishers (*t* = 2.98, uncorrected *p* < 0.005, cluster size >150). Small-volume correction at the peak voxel of the left CDn (10 mm radius) revealed that these effects were significant (Family-wise error corrected *p* = 0.03 at the cluster level). The results of the whole-brain analysis are presented in Table S2. The extraction of the CDn cluster displayed in Fig. 2D was performed using the bspmview toolbox (http://www.bobspunt.com/bspmview/), and the cluster was plotted on a 3D image of the basal ganglia (caudate and putamen) using the Multicolor toolbox (http://www.cns.atr.jp/multi_color/). The 3D image of the basal ganglia was constructed using the WFU_PickAtlas toolbox (http://fmri.wfubmc.edu/software/pickatlas).

### Approval and informed consent

Both behavioural and MRI studies were conducted at the Brain Science Institute of Tamagawa University. The study protocol was approved by the Tamagawa University Brain Science Institute Ethics Committees, and all experiments were conducted in accordance with the approved protocol, which met the requirements of the Declaration of Helsinki. Written informed consent was provided by each participant prior to participation in the study.

## Electronic supplementary material

Supplementary Methods and Analysis

Dataset
