# Supplementary material for: Behavioural Differences and Neural Substrates of Altruistic and Spiteful Punishment
Source: Sci Rep. 2017 Nov 7;7:14654. doi: 10.1038/s41598-017-15188-w (PMC5676782; doi:10.1038/s41598-017-15188-w)
Supplement: Supplementary file 1 — Supplementary Methods and Analysis [file 41598_2017_15188_MOESM1_ESM.pdf]

Supplementary Methods and Analyses for

Behavioural Differences and Neural Substrates of Altruistic and Spiteful Punishment

Toshio Yamagishi[1,2*], Yang Li[2,3], Alan S. R. Fermin[2], Ryota Kanai[4], Haruto Takagishi[2], Yoshie Matsumoto[2], Toko Kiyonari[5], and Masamichi Sakagami[2]

[1] Graduate School of International Corporate Strategy, Hitotsubashi University

[2] Brain Science Institute, Tamagawa University

[3] Department of Psychology, University of Melbourne

[4] Department of Neuroinformatics, Araya Inc

[5] School of Social Informatics, Aoyama Gakuin University

*Corresponding author: Toshio Yamagishi, Graduate School of International Corporate Strategy, Hitotsubashi University, 2-1-2 Hitotsubashi, Tokyo 101-8439, Japan. E-mail: yamagishitoshio@gmail.com

This PDF file includes:

Supplementary Methods

Fig S1, Table S1 – S2

Supplementary Data Analysis

Supplementary Methods

Demographic Constitution of the Sample Used in the Analysis

Fig S1 shows the demographic constitution of the sample of 453 participants whose data were used in the analysis.

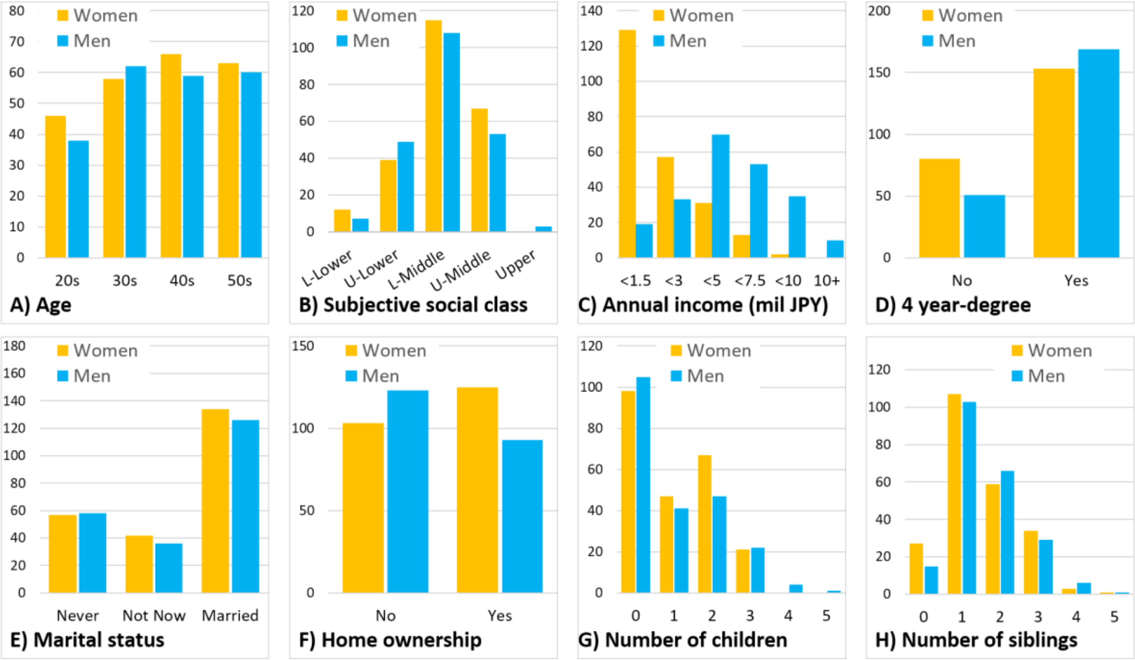

Fig S1. Major demographic constitution of the sample used in the analysis. The number of participants in some panels is less than 453 due to missing responses. Shown are (A) age, (B) subjective social class, (C) annual income in millions of Japanese Yen (JPY; N = 452), (D) completed 4-year degree, (E) marital status, (F) home ownership (N = 444), (G) number of children, and (H) number of siblings (including deceased; N = 451).

Games Included in the Measure of the Overall Pro-social Behaviour

PDG1: Repeated one-shot prisoner's dilemma game. PDG1 was conducted in October of 2012 through February of 2013. The game was repeated nine times, each time with a different partner. The participants were endowed with JPY 300, 800, or 1,500, which varied between games. Participants decided whether to provide this endowment to their partner or keep it for themselves. When the endowment was provided, the partner received twice the amount of the endowment. For example, in a

trial in which the size of the endowment was JPY 1,500, the partner received JPY 3,000 if the participant chose to give the endowment to the partner. Similarly, the participant received JPY 3,000 if the partner gave his/her endowment to the participant. When the participant did not provide and instead kept the endowment, he/she earned the kept amount. Of the nine games, each participant played the game according to the simultaneous protocol three times, as the first player in the sequential protocol three times, and as the second player three times. In the simultaneous protocol, the two players made decisions without knowing the other party's choice. In the sequential protocol, one player (first player) made a choice, following which the other player (second player) made a choice after being informed of the first player's choice.

The strategy method was used when the participant acted as the second player. That is, the participant decided whether to provide or keep his/her endowment twice in each trial, assuming once that the first player had decided to provide, and once that the first player had decided not to provide. The outcome of the game was determined based on the combination of the partner's actual choice and the participant's choice, given the partner's choice. Each participant played the game once for each combination of role and endowment size. The participants were instructed that three out of the nine games would be selected for actual payment. When all participants finished all nine games, one game was randomly selected from each stake size and used for actual payment. Each participant was randomly matched with another participant for each of the three games and paid for each game according to their actual choices.

Behavioural pro-sociality was defined based on the proportion of games in which an endowment was given to the randomly matched partner in the simultaneous and second-player sequential games in which the first had player cooperated. This excluded the participant's response to the first player's defection in the second player sequential games, as only very few of the participants cooperated in these games.

PDG2: One-shot, simultaneous prisoner's dilemma game. We used a one-shot PDG with the simultaneous protocol. PDG2 was conducted in September and October of 2013. The participants were endowed with JPY 1,000 and decided how much of the endowment to provide to their partner in increments of JPY 100. When the endowment was provided, the partner received twice the amount of the endowment. For example, when the participant provided JPY 300, the partner received JPY 600. Similarly, the participant received JPY 1,200 if the partner provided JPY 600. The portion of the endowment the participant did not provide was his or hers to keep. We used the proportion of endowment the participant provided to his or her partner as an indicator of behaviouralpro-sociality.

DG1: One-shot dictator game. Both DG1 and DG2 were conducted in April through June of 2013. All participants were instructed that pairs would be formed consisting of one dictator (referred to as the decider) and one recipient. However, all participants played the game as first players and decided how to divide the endowment of JPY 1,000. To make the payment consistent with the instructions, we paid each participant twice, once as a dictator and once as a recipient of a randomly matched dictator. The participants provided their behavioural choices on a decision sheet. We doubled the proportion that the participant allocated to his or her partners as an indicator of behaviouralpro-sociality in DG1. When the doubled mean proportion exceeded 1, we

set the participant’s pro-sociality indicator in the dictator game at 1, based on the assumption that giving one-half of the endowment is mostly pro-social. An additional analysis with the original score rather than the truncated score did not affect the results.

DG2: Repeated one-shot dictator game. Following DG1, the participants played similar games six times as a dictator, with a different recipient each time. The size of the endowment varied each time, ranging from JPY 300 to JPY 1,300 (300, 400, 600, 700, 1,200, and 1,300). Participants were told that they would play the game several times but were not told how many times they would play the game. All participants made allocation decisions as the dictator in each game, expecting that two trials would be selected for actual payment. They were further informed that in one of the two games they would receive the money they allocated to themselves as the dictator, and that in the other game they would receive the money that the matched participant allocated to them. The participants were paid according to this scheme. We doubled the mean proportion that the participant allocated to his or her partners as an indicator of behavioural pro-sociality in DG2. When twice the mean proportion exceeded 1, we set the participant’s pro-sociality indicator in the dictator game at 1, assuming that giving one-half of the endowment is mostly pro-social. An additional analysis with the original score rather than the truncated score did not affect the results.

SDG1 and SDG2: One-shot n-person social dilemma game. SDG2 is an exact replication of SDG1. SDG1 was conducted in September and October of 2013, and SDG2 was conducted in September through December of 2014. Participants were told that they would play the game in a group of which the actual size was not conveyed. The instruction was written for a 10-person group, but the participants were told that the actual group size could vary. The game was played once, and participants were paid based on their earnings in the game. Each participant was given an endowment of JPY 1,000 and decided how much to provide to produce a public good in increments of 100 JPY. The sum of the provided money for the public good was doubled and equally allocated to all members regardless of their provision level. We used the proportion of the endowment that the participant provided as an indicator of behaviouralpro-sociality in the social dilemma game.

TG: The responder’s behaviour in the one-shot trust game. The TG was conducted in December of 2013 through February of 2014. The TG was played between two randomly matched participants: a trustor and a responder. The trustor was provided with JPY 1,000 by the experimenter and decided how much to transfer to the responder in increments of JPY 100. The transferred money was then tripled and provided to the responder. The responder then decided how to transfer back to the trustor. The endowment of JPY 1,000 was provided only to the trustor and not to the responder, which differs from the standard version in which both players receive the same amount of endowment money. We introduced this feature to help the older non-student participants to clearly understand that the back transfer of half of what they had received as a responder resulted in a fair outcome when they were fully trusted by the trustor. Note that this is not the case in the standard trust or investment game. For example, when the trustor transfers JPY 1,000 and the responder returns JPY 1,500 or half of 3 times 1,000, the trustor receives JPY 1,500, and the responder receives JPY 1,500 plus the endowment of JPY 1,000. In this standard trust game, fairness depends on whether

players focus only on the transferred money or include the original amount given to the responder. All participants were told that they would play the game twice, each time with a different partner, and that their role would change. After they had read the instructions, they were all assigned the role of the trustor and decided how much of the JPY 1,000 to transfer to the responder. The first game ended when all participants made their decisions as trustors. Next, participants were told that they would play the game again with a different partner and in the role of the responder. In the second game, participants made decisions as responders using the strategy method. That is, they were asked to indicate the amount of money they would transfer back to the trustor in increments of 10% of the tripled money for each of the possible decisions of the matched trustor (when they transferred JPY 100, 200, 300, 400, 500, 600, 700, 800, 900, or 1,000). Finally, pairs of participants were formed randomly, and one of each pair was randomly chosen as a trustor and the other as responder. Each participant's earnings as a trustor depended on the money he/she had provided in the first game and the money that the randomly matched responder chose in response to the corresponding condition in the second game. We doubled the mean return proportion as an indicator of behavioural pro-sociality in the trust game. When the doubled mean return proportion exceeded 1, we set the participant's pro-sociality indicator in the trust game at 1, assuming that returning one-half of the endowment is mostly pro-social. An additional analysis with the original score rather than the truncated score did not affect the results. We used the mean return proportion of the tripled money when the first player transferred more than that of the endowment as an indicator of behavioural pro-sociality in the trust game.

Table S1. Correlations between the seven economic games and their factor loadings on the first factor

	PDG2	SDG1	SDG2	DG1	DG2	TG	Factor loadings
PDG1	.475	.440	.445	.476	.500	.512	0.607
PDG2		.691	.591	.423	.520	.537	0.762
SDG1			.647	.414	.493	.481	0.736
SDG2				.433	.484	.453	0.719
DG1					.722	.540	0.695
DG2						.611	0.783
TG							0.725

All correlations are significant at $\alpha = .0001$.

Punishment Games

TPPG: One-shot third-party punishment game. A total of 470 participants played the TPPG between May and July of 2014. Participants were told that they would play the game several times, and would be assigned one of three roles in each game: the "distributor" role, the "recipient" role, or the "observer" role. In each game, the distributor and the recipient played a dictator game, and the observer observed the outcome of the dictator game. Specifically, the distributor was endowed with JPY 1,500 and distributed the money between him/herself and a recipient in increments of JPY 100, and the recipient earned whatever amount the distributor assigned to him/her. The observer was informed of the distribution of JPY 1,500 between the distributor and the recipient, and decided by how much to reduce the earnings of the distributor in increments of JPY 100. The observer had to pay for the reduction in the distributor's earnings (i.e., the portion of the JPY 1,500 the distributor allocated to him/herself) at a rate of one quarter of the reduction amount. For example, the observer had to pay JPY 25 (50, 75, …) to reduce the distributor's earnings by JPY 100 (200, 300, …). The maximum amount of the observer's payment was JPY 375, which reduced the distributor's earnings by JPY 1,500. The strategy method was used for the observer's decision. That is, the observer was asked to decide how much to spend to reduce the distributor's earnings for each of the possible distributions of JPY 1,500. For example, the observer decided by how much to reduce the distributor's earnings if he/she distributed all JPY 1,500 to him/herself and nothing to the recipient: if he/she distributed JPY 1,400 to him/herself and JPY 100 to the recipient; if he/she distributed JPY 100 to him/herself and JPY 1,400 to the recipient; if he/she distributed nothing to him/herself and the entire JPY 1,500 to the recipient.

After all participants (4-10 per experimental session) finished their decisions in all three roles, each participant's role to be used for payment was randomly assigned, together with two other participant roles. Each participant was paid according to what happened in the trio. For example, when a participant was assigned an observer role, and the randomly matched distributor allocated JPY 500 to a recipient, the observer's (i.e. the participant's) earnings were dependent on how much he/she spent on reducing the distributor's earnings. That is, if he/she spent JPY 100 to reduce the distributor's earnings by JPY 400, he/she lost JPY 100 from his/her cumulative earnings in the day's tasks. When a participant was assigned the distributor's role, and decided to take JPY 1,200 for him/herself while giving the matched recipient JPY 300, and the randomly matched observer decided to reduce the matched distributor's earnings by JPY 600, the participant would earn JPY 600 (1,200 - 600). When the participant was assigned the recipient role and the randomly matched distributor gave him/her JPY 200, the participant's earnings were 200 JPY, regardless of what the randomly matched observed chose to do. Each participant first played the role of an observer, followed by the role of a distributor, and, finally, the role of a recipient.

In this TPPG, the observer spent the cost of punishment from his/her cumulative earnings (that is, the money he/she earned as a show-up fee and from other tasks). Immediately after finishing this no-endowment version of the TPPG, we conducted another version in which we provided the observer a JPY 500 endowment for punishment (payment for reducing the distributor's earnings). The correlation of *p*TPPG in the two versions was high (r = .860, P < .0001). We report here the results of the non-endowment TPPG. The findings of the non-endowment version of the TPPG were

mostly identical to those obtained using the endowment version of the game.

UG: One-shot ultimatum game. The UG was conducted with 471 participants between December of 2013 and February of 2014. Participants were instructed that they would play the same game twice, once as a proposer and once as a responder. All participants played the first game as a proposer, and then played the second game as a responder. As proposers, participants decided how much of an endowment of JPY 1,500 to provide to a randomly matched responder, in increments of JPY 100. In the second game, participants decided whether to accept or reject each of 16 possible proposals (JPY 0 to JPY 1500) made by a newly matched proposer. When all participants had played the game in both roles, random pairs were formed twice: once where the participant was the proposer and once as the responder. Each participant was paid according to the choice made in each pairing. We used the proportion of unfair offer rejection (JPY 100, 200, 300, 400 or 500) when participants played the role of the responder as a measure of *r*UG. Note that we excluded the participant's response to the proposal that would give him/her JPY 0, because rejecting that proposal cost nothing (zero) to the responder and thus did not meet the definition of a costly punishment. Eighty-six of the 129 participants who did not reject any unfair proposal rejected the proposal of zero to the responder, and the remaining 43 did not reject this extremely unfair proposal even though rejection of the proposal cost them nothing. The 86 costless UG rejecters were extremely low on behavioural pro-sociality (M = -0.632 ± 0.367) when compared with the 43 costless non-rejecters (M = 0.397 ± 0.381; t (68) = 4.80, $P < .0001$). The former were regarded as rational pro-self individuals who are indifferent to the other players' welfare or fairness of the distribution. The latter group seemed to include pacifists who were concerned with the other players' welfare (a high level of behavioural pro-sociality) and refrained from hurting other players for any reason (not rejecting unfair offers even when it is costless to do so).

Supplementary Data Analysis

Difference in the Ppreferences of Altruistic and Spiteful Punishers for the Four Outcomes in the One-shot PDG

In the post-experimental questionnaire of the one-shot PDG (PDG2), participants evaluated how satisfied they would be if the outcome of the game was each of the following four possible combinations of their own choice and the partner's choice: (CC) mutual cooperation outcome in which both players cooperated; (CD) "sucker's outcome" in which the participant cooperated but the partner defected; (DC) unilateral exploitation outcome in which the participant defected and the partner cooperated; (DD) mutual defection outcome in which both defected. Participants evaluated how satisfied they would be on a 7-point scale ranging from (1) "extremely unpleasant" to (7) "extremely happy". The two types of punishers differed in their evaluations of two of the four outcomes. First, spiteful punishers preferred (M = 4.325 ± 0.323, 95% confidence interval) the DC outcome of unilaterally exploiting the partner (preference for positive inequality or spite) more strongly than altruistic punishers (M = 3.723 ±

0.294); and the difference was highly significant ($t(249) = 2.73$, $P = .007$). The two types of punishers also differed in their evaluation of the CD outcome of being exploited by the partner (preference for negative inequity). Spiteful punishers were less aversive to exploitation (M = 2.228 ± 0.205) than altruistic punishers (M = 1.964 ± 0159; $t(249) = 2.05$, $P = .042$). The two types of punishers did not differ from each other in their evaluation of the CC outcome (spiteful punisher M = 5.807 ± 0.216; altruistic punisher M = 5.825 ± 0.179) of mutual cooperation or the DD outcome of mutual defection (spiteful punisher M = 4.403 ± 0.196; altruistic punisher M = 4.069 ± 0.180).

Table S2. Results of whole-brain analyses

Anatomical location	L/R	MNI coordinates x	y	z	Cluster size	t-value	Z-value	Whole-brain uncorrected	SVC FWE (10-voxel radius, cluster level)
Spiteful Punisher > Altruistic Punisher									
Lingual Gyrus	L	-16	-94	-3	833	3.74	3.68	0.000	0.014
Cuneus	L	-20	-104	10		3.11	3.08	0.001	
Middle Occipital Gyrus	L	-21	-96	8		3.10	3.06	0.001	
Insula	L	-42	-33	22	231	3.26	3.22	0.001	0.033
Transverse Temporal Gyrus	L	-39	-26	12		2.83	2.80	0.003	
Caudate	L	-6	8	2	201	2.98	2.95	0.002	0.030
Spiteful Punisher < Altruistic Punisher									
Middle Frontal Gyrus	L	-39	2	51	152	3.21	3.17	0.001	0.038

MNI: Montreal Neurological Institute; SVC: small-volume corrected; FWE: family-wise error.
